# Grape Seed Proanthocyanidin Extract Improves Growth Performance and Protects Against Hydrogen Peroxide-Induced Oxidative Stress to the Liver and Intestine in Weaned Hyla Rabbits

**DOI:** 10.3390/ani15030327

**Published:** 2025-01-24

**Authors:** Maohua Gong, Lei Liu, Fuchang Li, Jiali Chen

**Affiliations:** Key Laboratory of Efficient Utilization of Non-Grain Feed Resources (Co-Construction by Ministry and Province), Ministry of Agriculture and Rural Affairs, Shandong Provincial Key Laboratory of Animal Nutrition and Efficient Feeding, Department of Animal Science, Shandong Agricultural University, Tai’an 271017, China; ssgmh1996@163.com (M.G.); leiliu@sdau.edu.cn (L.L.)

**Keywords:** antioxidant capacity, H_2_O_2_, oxidative stress model, proanthocyanidin, weaned rabbits

## Abstract

Three experiments were conducted to investigate the effects of grape seed proanthocyanidin extract (GSPE) on the growth performance of weaned Hyla rabbits and explore its protective effects against oxidative stress in the liver and intestine by establishing a hydrogen peroxide (H_2_O_2_)-induced oxidative stress model. In Exp.1, ninety-six weaned rabbits were used to evaluate the effects of dietary GSPE level on growth performance, and the results showed that a 400 mg/kg GSPE addition increased the feed conversion ratio and liver coefficient and promoted cholesterol metabolism. Exp.2 was conducted to explore the H_2_O_2_ concentration required to establish an oxidative stress model, indicating that the model could be established by an intraperitoneal injection of 10% H_2_O_2_ at a dosage of 1 mL/kg body weight. In Exp.3, seventy-two weaned rabbits were used to investigate the protective effects against H_2_O_2_-induced oxidative stress in the liver and intestine. Our findings showed that 400 mg/kg GSPE supplementation could alleviate the adverse effects of H_2_O_2_ injection on the antioxidant capacity in the liver and intestine as well as liver morphology. Therefore, an addition of 400 mg/kg GSPE could improve growth performance and alleviate H_2_O_2_-induced adverse effects on the liver and small intestine by enhancing the antioxidative capacity in weaned Hyla rabbits.

## Simple Summary

Weaning is one of the most stressful events in a rabbit’s life, which could induce oxidative stress, leading to growth retardation. The liver and intestine are the body’s most important metabolic and digestive organs, respectively. However, these organs are the most sensitive to oxidative stress and highly susceptible to injury when oxidative stress occurs. Because using antibiotics as growth promoters is prohibited, it is essential to identify new antioxidants that can promote growth performance and protect rabbits against the damage caused by weaning-induced oxidative stress. Proanthocyanidins are naturally polyphenolic compounds with strong free radical scavenging ability and antioxidant properties, and they are especially abundant in grape seeds. However, there is limited research on the effects of grape seed proanthocyanidin extract (GSPE) on the growth performance of weaned meat rabbits and their role in alleviating oxidative stress in the liver and intestine. Our findings showed that supplementation with 400 mg/kg GSPE could improve growth performance and alleviate hydrogen peroxide-induced adverse effects on the liver and small intestine through enhancing the antioxidative capacity in weaned Hyla rabbits, providing support for the application of GSPE in rabbit farming.

## 1. Introduction

Reactive oxygen species (ROS), including hydrogen peroxide (H_2_O_2_) and superoxide, are continuously produced from oxygen (O_2_) during aerobic metabolism and various pathogenic processes [1]. Oxidative stress arises when the balance between ROS production and the body’s antioxidant defense mechanisms is disrupted [2]. This imbalance can trigger a cascade of reactions that damage lipids, proteins, and/or DNA, potentially leading to the development of numerous diseases [3]. The liver and intestine are the body’s most important metabolic and digestive organs, respectively. Meanwhile, they are especially vulnerable to oxidative stress due to the high number of mitochondria in their cells, which are the primary sites of ROS production [4,5,6]. Weaned rabbits become extremely vulnerable to oxidative stress due to the alteration of feed and environment [7]. Numerous studies have shown that oxidative stress can impair liver and intestinal morphology and function, leading to increased hepatic cell apoptosis and intestinal barrier permeability and ultimately resulting in growth retardation in rabbits [8,9]. Because using antibiotics as growth promoters is prohibited, it is essential to identify new antioxidants that protect rabbits against the damage caused by ROS.

Proanthocyanidins are naturally polyphenolic compounds with strong free radical scavenging ability and antioxidant properties. They have gained increasing interest due to their non-toxicity, bioavailability, and ability to provide multi-organ protection against toxicity induced by drugs and chemicals [10]. Grape seeds are the most abundant source of proanthocyanidins. Fawzia et al. [11] indicated that dietary 300 mg/kg grape seed extract containing 41.6% proanthocyanidins increased body weight (BW) and feed conversion ratio (FCR), enhanced plasma antioxidant enzyme activities, and reduced plasma malonaldehyde (MDA) concentration in rabbits. Grape seed proanthocyanidin extract (GSPE) was also found to play a beneficial role in the enhancement of liver function of rabbits in a heat stress-induced oxidative stress model [12]. Similarly, 250 and 500 mg/kg GSPE relieved Aflatoxin B1 (AFB_1_) exposure-induced adverse effects on growth performance and liver histopathological lesions via enhancing serum and hepatic antioxidant capacity [13]. A previous study in weaned piglets showed that a dietary addition of 50–150 mg/kg GSPE consisting of 79.28% oligomeric procyanidins enhanced antioxidant enzyme capacities and antioxidant-related gene expressions and decreased MDA concentration in the intestine [14]. Zheng et al. [15] found that 30–120 mg/kg GSPE (containing 86.81% proanthocyanidin oligomers, 2.41% epicatechin, 1.52% catechin, and 0.98% proanthocyanidin B2) decreased the feed-to-gain ratio (F/G) and elevated serum total superoxide dismutase (T-SOD) and glutathione peroxidase (GSH-Px) activities in growing pigs. However, there is limited research on the effects of GSPE on the growth performance of weaned meat rabbits and their role in alleviating oxidative stress in the liver and intestine.

H_2_O_2_ injection is a widely used procedure to establish the oxidative stress model in animals [16,17]. Hence, three experiments were conducted in this study to investigate the effects of GSPE on the growth performance of weaned meat rabbits and explore its role in alleviating oxidative stress in the liver and intestine by establishing an H_2_O_2_-induced oxidative stress model, providing support for the application of GSPE in rabbit farming.

## 2. Materials and Methods

### 2.1. Animals and Treatments

In Exp.1, a total of 96 weaned Hyla rabbits (35 d of age) were randomly assigned to four treatment groups with 6 replicates per treatment and 4 rabbits per replicate. The rabbits in the four groups were fed a basal diet supplemented with 0, 200, 400, or 800 mg/kg GSPE, denoted as G0, G200, G400, and G800, respectively. The individual fasting BWs at the start and end days of the trial, the daily feed intake per cage, and the number of deaths were recorded. Finally, the average daily gain (ADG), average daily feed intake (ADFI), F/G, and survival rate were calculated. The trial lasted for 35 days.

In Exp.2, to explore the concentration of H_2_O_2_ required to establish the oxidative stress model, sixty-four Hyla rabbits of 56 d age were randomly allocated to one of four treatment groups (8 replicates each group and 2 rabbits per group): (1) Blank, rabbits not injected with sterile saline (SS) or H_2_O_2_; (2) SS, rabbits injected with 0.9% SS intraperitoneally; (3) 5% H_2_O_2_, rabbits injected with 5% H_2_O_2_ intraperitoneally; (4) 10% H_2_O_2_, rabbits injected with 10% H_2_O_2_ intraperitoneally. The trial lasted for 13 days, consisting of a 6-day adaptive period and a 7-day formal experiment period. The SS or H_2_O_2_ was administered once on the first day of the formal experiment. The intraperitoneal injection dosage of SS or H_2_O_2_ in this study was 1 mL/kg BW. The injection dosage, frequency, and duration of the trial referred to the results in Yin et al. [17]. All the rabbits were fed the same basal diet during the formal experiment period, and the ADF, ADFI, F/G, and survival rate were calculated according to the method in Exp.1.

In Exp.3, seventy-two weaned Hyla rabbits of 35 d age were randomly divided into three treatment groups (6 replicates with 4 rabbits each) as follows: control group, H_2_O_2_ group, and H_2_O_2_+GSPE group. The rabbits in the control and H_2_O_2_ groups were fed the basal diet, whereas the rabbits in the H_2_O_2_+GSPE group were fed the basal diet supplemented with 400 mg/kg GSPE according to the results of Exp1. The trial lasted for 35 days. On day 29 of the trial, the rabbits in the H_2_O_2_ group and H_2_O_2_+GSPE group were injected with 10% H_2_O_2_ (1 mL/kg BW) intraperitoneally according to the results of Exp.2, while the rabbits in the control group were administered with an equivalent volume of 0.9% SS.

All the rabbits used in this study were purchased from Tai’an Xinglong Rabbit Farming (Tai’an, China). The basal diet (Table 1) used in the trials was formulated in line with the nutrient requirements recommended by the Nutrition Requirements of Meat Rabbits (NY/T 4049-2021, Ministry of Agriculture of China), and the GSPE (CAS 4852-22-6, purity ≥ 95%) was purchased from Solarbio Science & Technology Co., Ltd. (Beijing, China). All rabbits were housed in cages and were provided with ad libitum access to fresh water and feed during the trials. The lighting was controlled under a 12 h light and 12 h dark condition with room temperature between 20 and 23 °C.

### 2.2. Sampling Procedure

In Exp.1, at the end of the trial, eight rabbits from each group, with BWs closest to the average, were selected for collecting blood by heart punctures [8]. The serum was obtained after centrifugation for 15 min at 3000× *g* and immediately stored at −20 °C for further analysis. Subsequently, the rabbits were slaughtered after being euthanized. The full eviscerated weight and half eviscerated weight were recorded to calculate the full eviscerated weight ratio and half eviscerated weight ratio, respectively, as described by Kong et al. [18]. Additionally, the thymus, spleen, liver, and kidney were weighed to calculate the ratios of individual organs over BW accordingly.

In Exp.2, seven days after H_2_O_2_ injection (day 7 of the formal experiment period), one rabbit from each replicate with similar weight to the replicate average was selected for blood sampling to obtain the serum. The procedure was the same as that in Exp.1.

In Exp.3, on day 35 of the trial (seven days after H_2_O_2_ injection), eight rabbits per group with a similar BW to the average group weight were chosen and slaughtered immediately after deep anesthesia. After evisceration, the small intestine was dissected according to previous study [19]. The segments (2~3 cm) of duodenum, jejunum, and ileum were immediately cut and rinsed by sterile saline. One part of the intestinal tissues was fixed in 4% paraformaldehyde for morphological examination, and another part was stored at −80 °C after being snap-frozen in liquid nitrogen. Additionally, liver samples weighing about 5 g were promptly obtained from the central region of the hepatic lobule. A portion of these samples was rapidly frozen using liquid nitrogen and subsequently stored at −80 °C for future analysis, while the remaining portion was fixed in a 4% paraformaldehyde solution at room temperature.

### 2.3. Determination of Serum Biochemical Indicators and MDA

Serum total protein (TP), urea, albumin (ALB), triglycerides (TG), total cholesterol (TCHO), calcium (Ca), phosphorus (P), alkaline phosphatase (ALP), and MDA were determined using the commercial kits purchased from Nanjing Jiancheng Bioengineering Institute (Nanjing, China).

### 2.4. Measurements of Intestinal and Liver Morphology

After 24 h fixation in 4% paraformaldehyde solution, intestinal and liver tissue samples were dehydrated through graded levels of ethyl alcohol and embedded in liquid paraffin according to conventional paraffin-embedding protocol [20]. Then, paraffin wax embedded tissues were cut into 5 µm sections using a semi-automatic microtome (Leica Co., Wetzlar, Germany) and stained with hematoxylin and eosin (H&E). Photomicrographs were obtained using an Olympus BX51 microscope (Tokyo, Japan). Intestinal villus height (VH) and crypt depth (CD) were measured with JD801 morphologic image analysis software (Nanjing, China) as previously described [21], and the ratio of VH to CD (V/C) was also calculated.

### 2.5. Determination of Antioxidant Indexes in the Small Intestine and Liver

The intestinal and liver tissue samples were homogenized in ice-cold saline solution, and the supernatants were obtained after being centrifugated at 2500× *g* at 4 °C for 10 min. The levels of MDA, SOD, catalase (CAT), and glutathione (GSH) were determined using the commercial kits (Nanjing Jiancheng Bioengineering Institute, Nanjing, China) as described in detail in Chen et al. [19].

### 2.6. Statistical Analysis

The average cage data were used to assess the impacts on growth performance, and the individual rabbit was considered the experimental unit for the other variables using one-way ANOVA of SAS 9.4 (Institute Inc., Cary, NC, USA). Variations among the group means were compared using Duncan’s multiple comparisons. Results are expressed as means with the root mean square error (RMSE). A level of *p* < 0.05 was considered statistically significant.

## 3. Results

### 3.1. Effects of Dietary Supplementation with GSPE on the Growth Performance of Weaned Rabbits (Exp.1)

As shown in Table 2, the rabbits in the G400 group had a significantly lower F/G compared to the rabbits in the G0 group (*p* < 0.05). There were no significant differences in final BW (FBW), ADFI, and ADG among the four groups (*p* > 0.05). The survival rates were 79.8%, 79.8%, 91.7%, and 83.4%, respectively, for the G0, G200, G400, and G800 groups.

### 3.2. Effects of Dietary Supplementation with GSPE on the Slaughter Performance of Weaned Rabbits (Exp.1)

The data of the slaughter performance of meat rabbits are shown in Table 3. No significant differences were observed in the full eviscerated weight, half eviscerated weight, full eviscerated weight ratio, and half eviscerated weight ratio of rabbits among all the groups (*p* > 0.05).

### 3.3. Effects of Dietary Supplementation with GSPE on the Organ Coefficients of Weaned Rabbits (Exp.1)

The organ coefficients of meat rabbits are shown in Table 4. The organ coefficient of the liver was significantly higher in the G400 group than in the G0 group (*p* < 0.05). There were no significant differences among the four treatments concerning the ratios of thymus, spleen, and kidney weights over BW (*p* > 0.05).

### 3.4. Effects of Dietary Supplementation with GSPE on the Serum Biochemical Indicators of Weaned Rabbits (Exp.1)

The serum biochemical indicators of meat rabbits are shown in Table 5. The serum TCHO concentration in the G400 group was significantly lower than that in the G0 group (*p* < 0.05). There were no significant differences in the serum concentrations of TP, UREA, ALB, TG, Ca, P, and ALP among the four groups (*p* > 0.05).

### 3.5. Effects of H_2_O_2_ Injection on the Growth Performance of Hyla Rabbits (Exp.2)

As shown in Table 6, SS injection had no significant effects on the growth performance of rabbits (*p* < 0.05), but 10% H_2_O_2_ injection significantly decreased the FBW, ADFI, and ADG and increased F/G ratio compared to the other three groups (*p* < 0.05). Additionally, the 5% H_2_O_2_ group had significantly lower ADG and a higher F/G ratio than the Blank and SS groups (*p* < 0.05). No rabbits died in the four treatments during the trial.

### 3.6. Effects of H_2_O_2_ Injection on the Serum MDA Concentration of Hyla Rabbits (Exp.2)

**Figure 1:**
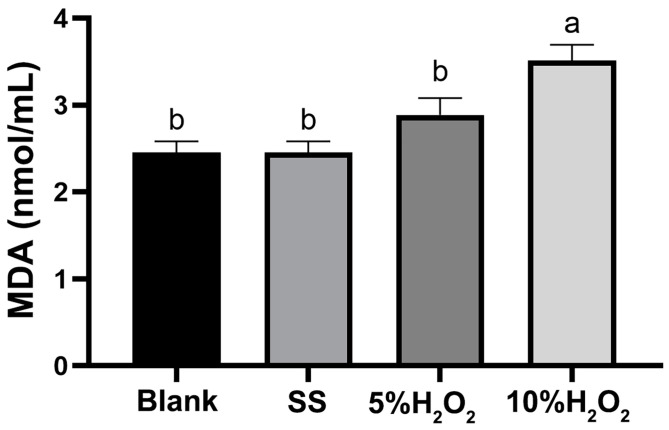
Effects of hydrogen peroxide (H_2_O_2_) injection on serum malondialdehyde (MDA) content of Hyla rabbits. Blank, rabbits not injected with sterile saline (SS) or H_2_O_2_ intraperitoneally; SS, rabbits injected with SS intraperitoneally; 5% H_2_O_2_, rabbits injected with 5% H2O2 intraperitoneally; 10% H_2_O_2_, rabbits injected with 10% H_2_O_2_ intraperitoneally. ^a,b^ Means with different lowercase letters differ significantly among treatments (*p* < 0.05). *n* = 8.

Figure 1 showed that 10% H_2_O_2_ injection had the highest serum MDA concentration, which was significantly higher than the other three groups (*p* < 0.05).

### 3.7. Effects of Dietary Supplementation with GSPE on Liver Morphology of Weaned Rabbits Challenged with H_2_O_2_ (Exp.3)

**Figure 2:**
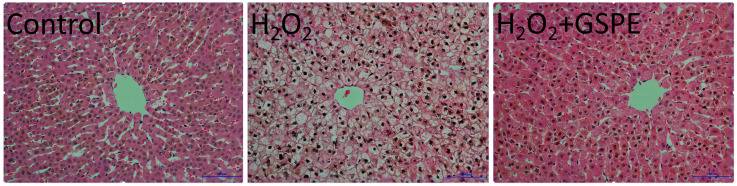
Effects of dietary supplementation with grape seed proanthocyanidin extract (GSPE) on liver morphology of weaned rabbits challenged with hydrogen peroxide (H_2_O_2_). Control, rabbits fed the basal diet; H_2_O_2_, rabbits fed the basal diet and given intraperitoneal injection of H_2_O_2_; H_2_O_2_+GSPE, rabbits fed the basal diet supplemented with 400 mg/kg and given intraperitoneal injection of H_2_O_2_.

As shown in Figure 2, the liver cells in the control group were structurally intact and arranged in an orderly manner to form liver cords, with a clear nucleus located centrally in the cell. Meanwhile, H_2_O_2_ injection resulted in liver cell swelling, vacuolization, and unclear cell spaces and liver cords, with the cell nucleus shifting or even disappearing. However, GSPE supplementation alleviated the adverse changes in liver morphology caused by the intraperitoneal injection of H_2_O_2_.

### 3.8. Effects of Dietary Supplementation with GSPE on Liver Oxidative Capacity of Weaned Rabbits Challenged with H_2_O_2_ (Exp.3)

**Figure 3:**
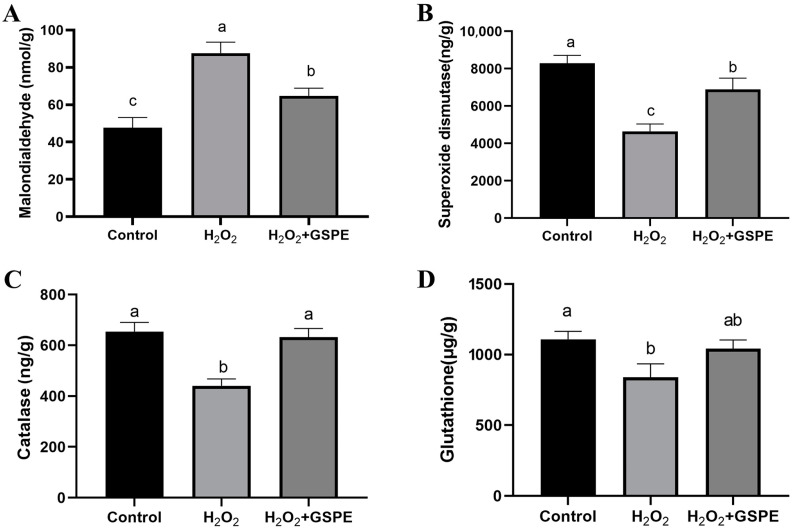
Effects of dietary supplementation with grape seed proanthocyanidin extract (GSPE) on liver oxidative capacity of weaned rabbits challenged with hydrogen peroxide (H_2_O_2_). (**A**) malondialdehyde; (**B**) superoxide dismutase; (**C**) catalase; (**D**) glutathione. Control, rabbits fed the basal diet; H_2_O_2_, rabbits fed the basal diet and given intraperitoneal injection of H_2_O_2_; H_2_O_2_+GSPE, rabbits fed the basal diet supplemented with 400 mg/kg and given intraperitoneal injection of H_2_O_2_. ^a,b,c^ Means with different lowercase letters differ significantly among treatments (*p* < 0.05). *n* = 8.

Relative to the control group, H_2_O_2_ injection significantly elevated the MDA concentration (*p* < 0.05) and significantly reduced the levels of SOD, CAT, and GSH in the liver (*p* < 0.05) (Figure 3). In H_2_O_2_-challenged rabbits, dietary GSPE supplementation significantly (*p* < 0.05) decreased MDA concentration and increased SOD and CAT activities in the liver. The rabbits in the H_2_O_2_+GSPE group had significantly higher MDA concentration and lower SOD activity than the rabbits in the control group (*p* < 0.05), and no significant differences were observed in the CAT and GSH activities between the two groups (*p* > 0.05).

### 3.9. Effects of Dietary Supplementation with GSPE on Small Intestinal Morphometric Measurements of Weaned Rabbits Challenged with H_2_O_2_ (Exp.3)

**Figure 4:**
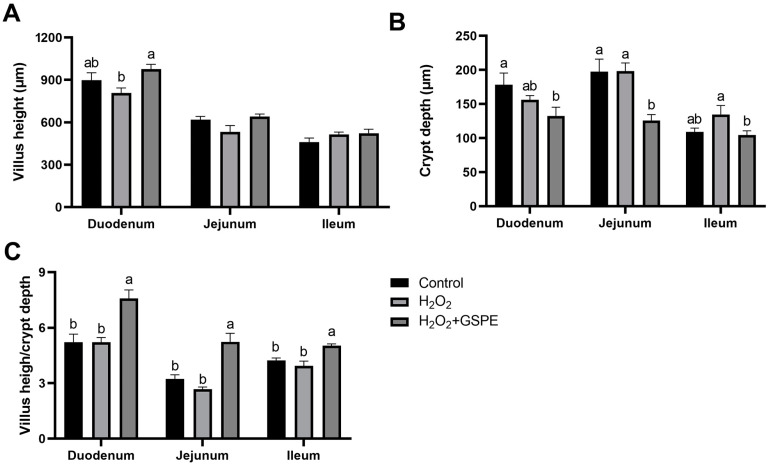
Effects of dietary supplementation with grape seed proanthocyanidin extract (GSPE) on small intestinal morphometric measurements of weaned rabbits challenged with hydrogen peroxide (H_2_O_2_). (**A**) Villus height; (**B**) crypt depth; (**C**) villus height/crypt depth. Control, rabbits fed the basal diet; H_2_O_2_, rabbits fed the basal diet and given intraperitoneal injection of H_2_O_2_; H_2_O_2_+GSPE, rabbits fed the basal diet supplemented with 400 mg/kg and given intraperitoneal injection of H_2_O_2_. ^a,b^ Means with different lowercase letters differ significantly among treatments (*p* < 0.05). *n* = 8.

Compared to the control group, H_2_O_2_ administration had no significant effects on the VH, CD, and V/C of duodenum, jejunum, and ileum (*p* > 0.05) (Figure 4). The rabbits in the H_2_O_2_+GSPE group had significantly higher duodenal VH and lower ileal CD than the rabbits in the H_2_O_2_ group (*p* < 0.05) and showed significantly lower duodenal CD than the rabbits in the control group (*p* < 0.05). Moreover, the H_2_O_2_+GSPE group had significantly lower jejunal CD (*p* < 0.05) and significantly higher V/C in the duodenum, jejunum, and ileum than the other two groups (*p* < 0.05).

### 3.10. Effects of Dietary Supplementation with GSPE on Small Intestinal Oxidative Capacity of Weaned Rabbits Challenged with H_2_O_2_ (Exp.3)

**Figure 5:**
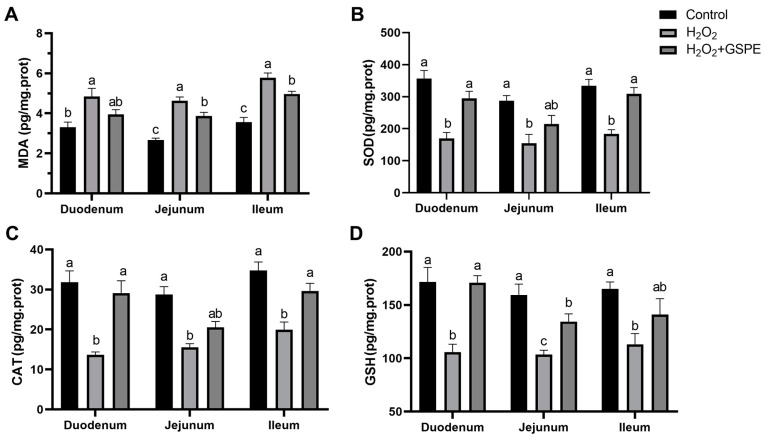
Effects of dietary supplementation with grape seed proanthocyanidin extract (GSPE) on small intestinal oxidative capacity of weaned rabbits challenged with hydrogen peroxide (H_2_O_2_). (**A**) MDA, malondialdehyde; (**B**) SOD, superoxide dismutase;(**C**) CAT, catalase; (**D**) GSH, glutathione. Control, rabbits fed the basal diet; H_2_O_2_, rabbits fed the basal diet and given intraperitoneal injection of H_2_O_2_; H_2_O_2_+GSPE, rabbits fed the basal diet supplemented with 400 mg/kg and given intraperitoneal injection of H_2_O_2_. ^a,b,c^ Means with different lowercase letters differ significantly among treatments (*p* < 0.05). *n* = 8.

Compared to the control group, H_2_O_2_ injection significantly increased the MDA concentration and inhibited the activities of SOD, CAT, and GSH in the duodenum, jejunum, and ileum of rabbits (*p* < 0.05) (Figure 5). In H_2_O_2_-challenged rabbits, dietary GSPE supplementation significantly decreased the MDA concentrations in the jejunum and ileum (*p* < 0.05) and significantly increased the SOD and CAT activities in the duodenum and ileum, as well as GSH activity in the jejunum of meat rabbits (*p* < 0.05). Additionally, the rabbits in the H_2_O_2_+GSPE group showed significantly higher MDA concentrations in the jejunum and ileum (*p* < 0.05) and significantly lower GSH activity in the jejunum relative to the control group (*p* < 0.05).

## 4. Discussion

In the present study, dietary supplementation with 400 mg/kg GSPE decreased the F/G ratio, but GSPE addition had no significant effects on the slaughter performance in meat rabbits. A previous study in broilers also showed that 7.5 and 15 mg/kg GSPE decreased the F/G ratio without affecting the eviscerated yield [22]. Additionally, 200 and 400 mg/kg GSPE supplementation was found to increase the FCR in goldfish [23]. Many studies have attributed the growth-promoting effects of GSPE to its antioxidant activity [24,25]. It is well known that weaning of rabbits offers nutritional and animal health benefits in rabbit production. But undeniably, weaning rabbits for does is one of the most stressful events, as rabbits often suffer from physiological, nutritional, and social stresses during the weaning progress. It was reported that the weaning process could induce abnormal behavioral traits and physiological responses, such as increased cortisol, CAT, and SOD levels [7]. An elevated blood cortisol level was usually associated with oxidative stress, and increasing antioxidative enzyme activities was a way to overcome the produced ROS [7,26]. Oxidative stress caused by weaning is a significant factor leading to growth retardation in livestock. Previous studies have shown that oxidative stress can damage the intestinal mucosal barrier and inhibit gut enzyme activity, reducing nutrient digestion and absorption [27,28]. Moreover, oxidative stress also could induce liver injury and loss of liver function and thus suppress the secretion of growth and development-related hormones [29,30]. GSPE has been shown to possess a wide range of pharmacological and medicinal properties in mitigating oxidative stress. It has been proven that GSPE can alleviate exogenous (such as cisplatin, lipopolysaccharides, zearalenone, and H_2_O_2_) and endogenous (such as diabetes) oxidative stress [31,32,33,34]. Mu et al. [35] found that the supplementation of GSPE (0–40 mg/kg BW/day) linearly increased the liver weight of ram lambs, suggesting that GSPE potentially plays a crucial role in the metabolism of organisms. Similarly, 400 mg/kg GSPE supplementation significantly increased the liver coefficient of rabbits. These results suggest that the optimal level of GSPE supplementation in the diet promoted the development of liver in the rabbits. The liver plays a key role in the synthesis, transport, conversion, and excretion of cholesterol. In the present study, the serum TCHO of weaned rabbits was significantly decreased by dietary 400 mg/kg GSPE supplementation. Previous studies in rats have indicated that GSPE can promote lipid metabolism and inhibit fat deposition [36]. Feng et al. [37] also showed that adding 100 and 200 mg/kg GSPE to the diet reduced serum TCHO levels but increased serum high-density lipoprotein cholesterol (HDL-C) levels in pigs. HDL-C, produced by the liver, helps to transport excess cholesterol from peripheral tissues back to the liver, preventing cholesterol accumulation in the blood vessels [38]. Therefore, the decreased serum TCHO of rabbits in the G400 group might be related to the regulation of lipid metabolism by the GSPE, which needs to be studied further. However, exposure to higher concentrations of GSPE could result in ROS generation and cell death [39], which might be the reason for the decreased liver coefficient and increased serum TCHO level in the G800 group. The above results also indicated the importance of optimizing the concentration of GSPE for application to avoid potential harmful pro-oxidant effects in animal feed.

H_2_O_2_ is one of the major and the most stable ROS in the redox regulation of biological activities. Excessive H_2_O_2_ is harmful to almost all cell components, so it is often used to establish the oxidative stress model [16,17]. In this study, 10% H_2_O_2_ injection showed an adverse effect on the growth performance of rabbits. Similar results have also been reported in a 10% H_2_O_2_-induced oxidative stress model [17,40]. Additionally, increased MDA concentrations in the serum, liver, and small intestine were found in the rabbits injected with 10% H_2_O_2_ in the current study. MDA is usually considered a reliable biomarker of oxidative stress [41]. Therefore, a rabbit oxidative stress model was well established by an intraperitoneal injection of 10% H_2_O_2_ in the present study.

The liver and intestine are the organs that are most sensitive to oxidative stress and are highly susceptible to injury when the oxidative stress occurs [42]. In this study, the liver morphological changes of rabbits were induced by 10% H_2_O_2_ injection. A previous study in vitro showed that H_2_O_2_ treatment could result in the accumulation of the ROS through disrupting the intracellular oxidative homeostasis via inhibiting antioxidant enzyme activities, inducing the oxidative damage in HepG2 cells [43]. Reduced SOD, CAT, and GSH activities and elevated MDA level by H_2_O_2_ injection were also found in the current study, but GSPE supplementation could alleviate these changes to the levels observed in the control group. SOD and CAT play crucial roles in protecting the body from oxidative damage. SOD converts ROS into H_2_O_2_, while CAT is responsible for detoxifying H_2_O_2_, breaking it down into H_2_O and O_2_ [44]. GSH is a low molecular weight thiol-tripeptide capable of directly reacting with free radicals and peroxides to reduce oxidative stress and protect cells from damage [6]. It has been proven that PPARα, Nrf2/ARE, and PI3K/AKT signaling pathways are all involved in the protective effect of GSPE against liver oxidative damage [33,45]. Rajput et al. [13] found that 250 and 400 mg/kg GSPE decreased MDA concentration and increased T-SOD, CAT, GSH, and GSH-Px activities in the liver, alleviating the liver injury caused by the ABF_1_ of broilers. A study in Kunming mice also showed that 100 mg/kg GSPE could activate the Nrf2/ARE signaling pathways in the liver and protect against zearalenone-induced liver oxidative damage [34]. Therefore, GSPE supplementation could mitigate H_2_O_2_-induced liver injury by enhancing liver antioxidant capacity in meat rabbits. However, the mechanism by which GSPE alleviates H_2_O_2_-induced hepatic oxidative damage in rabbits still requires further investigation.

The crypt–villus architecture of the small intestine is essential for maintaining the structural integrity of the intestinal epithelium and preservation of gut homeostasis [46]. The V/C ratio is regarded as a valuable criterion for assessing the nutrient absorption capacity of the small intestine [18]. In the present study, 10% H_2_O_2_ injection was not found to significantly damage the crypt–villus architecture of the small intestine compared with the control group, although it disrupted the small intestinal redox homeostasis of rabbits. This might be attributed to the intestinal property of self-repair to maintain mucosal homeostasis [46], and 10% H_2_O_2_ injection once was not enough to induce extensive damage to intestinal morphology. However, GSPE supplementation showed beneficial effects to improve the small intestinal morphology of meat rabbits, especially under H_2_O_2_-induced oxidative stress conditions. Previous studies in pigs [14], broiler hens [47], and mice [48] have manifested that GSPE supplementation could improve intestinal health through enhancing antioxidant capacity and modulating gut microbiota. Consistently, enhanced SOD, CAT, and GSH activities and reduced MDA concentration in the small intestine were found in the H_2_O_2_-challenged rabbits fed the diet supplemented with GSPE. Therefore, GSPE supplementation could mitigate the H_2_O_2_-induced intestinal oxidative stress of the meat rabbits in this study.

## 5. Conclusions

Collectively, supplementation with 400 mg/kg GSPE could increase the FCR and promote the development of liver and cholesterol metabolism in weaned Hyla rabbits. The intraperitoneal injection of 10% H_2_O_2_ at dosage of 1 mL/kg BW decreased growth performance, induced oxidative stress of the liver and small intestine, and damaged the liver morphology in the rabbits, showing that this dosage could be used to establish a rabbit oxidative stress model. However, dietary 400 mg/kg GSPE supplementation alleviated H_2_O_2_-induced adverse effects on the liver and small intestine by enhancing the antioxidative capacity in weaned Hyla rabbits.

## Figures and Tables

**Table 1:** Composition and nutrient levels of the experimental diet.

Ingredients (%)	Content	Nutrient Levels ^2^ (%)	Content
Corn	17	Digestible energy (MJ/kg)	10.25
Rice husk	5	Crude protein	15.86
Wheat bran	20	Crude fiber	18.28
Soybean meal	14	Acid detergent fiber	19.98
Alfalfa meal	24	Neutral detergent fiber	36.25
Bean straw powder	16	Ether extract	3.71
Premix ^1^	4	Calcium	0.78
Total	100	Phosphorus	0.43

^1^ The premix provided the following per kg of diets: Vitamin D_3_ 2000 IU, Vitamin B_5_ 375 mg, Vitamin B_7_ 1.5 mg, Vitamin E 22.5 mg, Vitamin A 6000 IU, Vitamin K 15 mg, Vitamin B_2_ 45 mg, Vitamin B_3_ 375 mg, Vitamin B_1_ 15 mg, Vitamin B_6_ 15 mg, Vitamin B_12_ 0.15 mg, choline chloride 625 mg, Se 0.05 mg, Zn 50 mg, ferrous sulfate 100 mg, iodine 0.6 mg, methionine 1500 mg, lysine 1500 mg, diclazuril 5mg. ^2^ Nutrient levels were measured values.

**Table 2:** Effects of dietary supplementation with grape seed proanthocyanidin extract (GSPE) on the growth performance of rabbits.

Items ^1^	Treatment ^2^				RMSE	*p* Value
	G0	G200	G400	G800		
IBW (g)	800	797	801	809	21.10	0.756
FBW (g)	1853	1863	1832	1835	76.66	0.881
ADFI (g/d)	125.18	124.66	118.13	120.39	8.12	0.391
ADG (g/d)	30.10	30.47	29.45	29.31	1.98	0.715
F/G	4.16 ^a^	4.09 ^ab^	4.01 ^b^	4.11 ^ab^	0.08	0.043
Survival rate (%)	79.8	79.8	91.7	83.4	-	-

^1^ IBW, initial body weight; FBW, final body weight; ADFI, average daily feed intake; ADG, average daily gain; F/G, ratio of ADFI to ADG. ^2^ G0, G200, G400, and G800 were rabbits fed the basal diet supplemented with 0, 200, 400, and 800 mg/kg GSPE, respectively. ^a,b^ Means with different letters within a row differ (*p* < 0.05). *n* = 8.

**Table 3:** Effects of dietary supplementation with grape seed proanthocyanidin extract (GSPE) on slaughter performance of meat rabbits.

Items	Treatment ^1^				RMSE	*p* Value
	G0	G200	G400	G800		
Full eviscerated weight (g)	785	790	799	816	7.34	0.894
Full eviscerated weight ratio (%)	41.29	41.31	41.58	42.62	2.15	0.681
Half eviscerated weight (g)	872	871	894	897	8.27	0.918
Half eviscerated weight ratio (%)	45.85	45.58	46.50	46.84	2.28	0.764

^1^ G0, G200, G400, and G800 were rabbits fed the basal diet supplemented with 0, 200, 400, and 800 mg/kg GSPE, respectively. *n* = 8.

**Table 4:** Effects of dietary supplementation with grape seed proanthocyanidin extract (GSPE) on organ coefficients in meat rabbits.

Items	Treatment ^1^				RMSE	*p* Value
	G0	G200	G400	G800		
Thymus (g/kg)	2.56	2.31	2.48	2.26	0.24	0.117
Spleen (g/kg)	0.864	0.785	0.761	0.764	0.17	0.683
Liver (g/kg)	26.69 ^b^	28.41 ^ab^	33.65 ^a^	29.11 ^ab^	3.39	0.013
Kidney (g/kg)	7.22	6.52	7.13	6.86	0.88	0.519

^1^ G0, G200, G400, and G800 were rabbits fed the basal diet supplemented with 0, 200, 400, and 800 mg/kg GSPE, respectively. ^a,b^ Means with different letters within a row differ (*p* < 0.05). *n* = 8.

**Table 5:** Effects of dietary supplementation with grape seed proanthocyanidin extract (GSPE) on serum biochemical indicators in meat rabbits.

Items ^2^	Treatment ^1^				RMSE	*p* Value
	G0	G200	G400	G800		
TP (g/L)	57.15	59.28	55.75	56.42	3.82	0.429
UREA (g/L)	8.51	9.00	7.84	7.57	1.51	0.363
ALB (g/L)	30.55	32.17	31.57	30.82	1.97	0.492
TG (mmol/L)	0.66	1.20	0.70	0.82	0.58	0.393
TCHO (mmol/L)	1.93 ^a^	1.74 ^ab^	1.49 ^b^	1.68 ^ab^	0.20	0.012
Ca (mmol/L)	4.60	4.62	4.69	4.61	0.20	0.869
P (mmol/L)	3.06	3.28	3.11	3.13	0.50	0.738
ALP (U/L)	116.17	125.00	132.00	111.50	24.37	0.486

^1^ G0, G200, G400, and G800 were rabbits fed the basal diet supplemented with 0, 200, 400, and 800 mg/kg GSPE, respectively. ^2^ TP, total protein; UREA, urea; ALB, albumin; TG, triglycerides; TCHO, total cholesterol; Ca, calcium; P, phosphorus; ALP, alkaline phosphatase. ^a,b^ Means with different letters within a row differ (*p* < 0.05). *n* = 8.

**Table 6:** Effects of hydrogen peroxide (H_2_O_2_) injection on the growth performance of Hyla rabbits.

Items ^2^	Treatment ^1^				RMSE	*p* Value
	Blank	SS	5% H_2_O_2_	10% H_2_O_2_		
IBW (g)	1468	1474	1469	1466	30.77	0.961
FBW (g)	1681 ^a^	1688 ^a^	1656 ^a^	1612 ^b^	37.82	0.002
ADFI (g/d)	126.10 ^a^	125.72 ^a^	118.64 ^a^	105.28 ^b^	13.51	0.015
ADG (g/d)	30.38 ^a^	30.54 ^a^	26.73 ^b^	20.74 ^c^	3.41	<0.001
F/G	4.15 ^c^	4.12 ^c^	4.45 ^b^	5.10 ^a^	0.17	<0.001
Survival rate (%)	100	100	100	100	-	-

^1^ Blank, rabbits not injected with sterile saline (SS) or H_2_O_2_ intraperitoneally; SS, rabbits injected with SS intraperitoneally; 5% H_2_O_2_, rabbits injected with 5% H_2_O_2_ intraperitoneally; 10% H_2_O_2_, rabbits injected with 10% H_2_O_2_ intraperitoneally. ^2^ IBW, initial BW; FBW, final BW; ADFI, average daily feed intake; ADG, average daily gain; F/G, ratio of ADFI to ADG. ^a,b,c^ Means with different letters within a row differ (*p* < 0.05). *n* = 8.

## Data Availability

The original contributions presented in this study are included in the article. Further inquiries can be directed to the corresponding authors.
